# *Bmovo*-*1* Regulates Ovary Size in the Silkworm, *Bombyx mori*

**DOI:** 10.1371/journal.pone.0104928

**Published:** 2014-08-13

**Authors:** Renyu Xue, Xiaolong Hu, Guangli Cao, Moli Huang, Gaoxu Xue, Ying Qian, Zuowei Song, Chengliang Gong

**Affiliations:** 1 School of Biology *&* Basic Medical Science, Soochow University, Suzhou, China; 2 National Engineering Laboratory for Modern Silk, Soochow University, Suzhou, PR China; New Mexico State University, United States of America

## Abstract

The regulation of antagonistic OVO isoforms is critical for germline formation and differentiation in *Drosophila*. However, little is known about genes related to ovary development. In this study, we cloned the *Bombyx mori ovo* gene and investigated its four alternatively spliced isoforms. BmOVO-1, BmOVO-2 and BmOVO-3 all had four C2H2 type zinc fingers, but differed at the N-terminal ends, while BmOVO-4 had a single zinc finger. *Bmovo*-1, *Bmovo*-2 and *Bmovo*-4 showed the highest levels of mRNA in ovaries, while *Bmovo*-3 was primarily expressed in testes. The mRNA expression pattern suggested that *Bmovo* expression was related to ovary development. RNAi and transgenic techniques were used to analyze the biological function of *Bmovo*. The results showed that when the *Bmovo* gene was downregulated, oviposition number decreased. Upregulation of *Bmovo*-1 in the gonads of transgenic silkworms increased oviposition number and elevated the trehalose contents of hemolymph and ovaries. We concluded that *Bmovo*-1 was involved in protein synthesis, contributing to the development of ovaries and oviposition number in silkworms.

## Introduction

In *Drosophila*, isoforms of OVO-B and OVO-A are produced by two major classes of germline *ovo* transcripts driven by the *ovo-A* and *ovo-B* promoters [1], [2]. *Ovo-B* and *ovo-A* transcripts differ only in their short first exons; OVO-B has 374 fewer residues than OVO-A. *Ovo-B* mRNA encodes the OVO-B isoform from an AUG initiation codon in exon 2, and the *ovo-A* transcript encodes the longer OVO-A isoform. The OVO-B and OVO-A isoforms have a common C2H2 zinc-finger domain but different N-termini that include potential effector domains [3]. OVO-B and OVO-A are transcription factors with opposing regulatory activities that are required for female germline survival and oogenesis [4]. Four dominant-negative *ovo* alleles (*ovo^D^*) strongly suggest that the OVO-A N-terminal region is functionally important [2], [5]. All four *ovo^D^* mutations introduce novel in-frame AUG codons upstream of the *ovo-B* initiation codon that the result in *ovo-B* transcripts that encode slightly truncated *ovo-A* isoforms. OVO-B expression is high during oogenesis, but OVO-A is expressed very weakly and only in nearly mature follicles [2], [4].

OVO-B is a transcriptional activator, and OVO-A is a transcriptional repressor. OVO-B positively regulates the *ovarian tumor* (*otu*) promoter and OVO-A represses target promoters [2], [4], [6], [7]. During most of oogenesis, OVO-B is necessary and sufficient for female germline development. OVO-B isoforms supply *ovo* ^+^ function in the female germline and epidermis. OVO-A isoforms have dominant negative activity in both tissues and elevated expression of OVO-A causes maternal-effect lethality. The absence of OVO-A results in maternal-effect sterility, indicating that tight regulation of antagonistic OVO-B and OVO-A isoforms is critical for germline formation and differentiation [4].

*Ovo^+^* genes encode putative C2H2 zinc-finger transcription factors [3], [7]–[10] in flies, nematodes, mice and humans. In flies and mice, *ovo* mutants have sex-specific reproductive defects and poor hair formation [10]–[12], revealing OVO conservation of function over evolutionary distance. Besides *Drosophila*, the insect genomes of Lepidoptera, Coleoptera, Hymenoptera, Hemiptera, Diptera and Anoplura have *ovo*-like genes. *Drosophila* OVO is well characterized, but few studies have reported on OVO in insects other than in *Drosophila* [13].

The silkworm *Bombyx mori* is a silk-spinning insect. As a model of Lepidopteran, its genome sequence is established [14], [15], and genes related to growth, development, metamorphosis, immunologic response, and fibroin synthesis have been comprehensively studied. However, little is known about genes related to ovary development. In this study, to understand the molecular mechanisms of silkworm nutrient metabolism and ovary development, four alternatively spliced isoforms of *ovo* genes were characterized. In this study, four alternatively spliced isoforms of *Bmovo* genes were cloned and investigated, *Bmovo*-1, *Bmovo*-2 and *Bmovo*-4 showed the highest levels of mRNA in ovaries, while *Bmovo*-3 was primarily expressed in testes. BmOVO-1, BmOVO-2 and BmOVO-3 all had four C2H2 type zinc fingers, but differed at the N-terminal ends, while BmOVO-4 had a single zinc finger. We hypothesized that *Bmovo-1* might be involved in regulating protein synthesis. In the study, the transgenic and RNAi techniques were applied to upregulate and downregulate *Bmovo-1* in silkworm ovaries to explore the mechanism on ovary size, protein synthesis, nutrition transportation and oviposition number regulated by *Bmovo-1*.

## Materials and Methods

### RNA isolation and cDNA synthesis

Total RNA was isolated from silkworm tissues using total RNA Isolation Kits (TaKaRa, Dalian, China), followed by treatment with DNaseI. cDNA was synthesized by PrimeScript Reverse Transcriptase (TaKaRa), following standard instructions.

### Cloning and sequencing of *Bmovo* gene

*Bmovo* gene-specific primers (Table S1) with endonuclease sites were designed using hypothetical *Bmovo* cDNA sequences obtained through *in silico* cloning based on *Drosophila* OVO (DmOVO) protein sequences (GenBank accession no. NP_726971), using tBLASTn (http://www.ncbi.nlm.nih.gov/blast). Polymerase chain reaction (PCR) used synthesized cDNA from larval gonads as a template. Primers ovo-1/ovo-2, Bmsovo-BH/Bmsovo-M2 and Bmsovo-M/Bmsovo-HD were designed according to the hypothetical *Bmovo* gene cDNA sequence and were used to amplify the *Bmovo* gene. PCR products were subjected to agarose gel electrophoresis and recombinant plasmids were sequenced after PCR products were cloned into the vector pMD19-T (TaKaRa, Dalian, China). The sequences have been deposited in GenBank at accession no. GU477588 (*Bmovo-*1), HQ831344 (*Bmovo-*2), HQ831345 (*Bmovo-*3) and JQ665224 (*Bmovo-*4).

### Sequence analysis of *Bmovo*

A homology search and multiple alignments were carried out with BLAST (http://www.ncbi.nlm.nih.gov/BLAST) and Clustal W software [16]. A phylogenetic tree was constructed with MEGA5.0 using the neighbor-joining method [17] based on OVO protein sequences from GenBank. The validity of branches was tested by bootstrapping using 1000 replicates. Secondary structure, conserved motifs and protein function were predicted using the Conserved Domain Search Service program (http://www.ncbi.nlm.nih.gov/Structure/cdd/wrpsb.cgi), SMART software (http://smart.embl-heidelberg.de/).

### BmOVO-1 antibody preparation

Bmovo-1 PCR product (2.4 kb) was digested with restriction endonuclease of *Bam*HI and *Xho*I and ligated into the expression vector pET-28a (+) (Novagen, Darmstadt, Germany) to generate the recombinant plasmid pET-28a (+)-Bmovo-1. Fusion proteins were expressed in *Escherichia coli* strain BL21. Recombinant protein was purified with Ni-NTA agarose (Qiagen, Shanghai, China) and used to immunize Kunming mice (Soochow University, Suzhou, China) by subcutaneous injection. Antibody was confirmed by Western blotting. This study was carried out in strict accordance with the recommendations in the Guide for the Care and Use of Laboratory Animals of the National Institutes of Health. The protocol was approved by the Committee on the Ethics of Animal Experiments of Soochow University of China (Permit Number: 201304326). All the experimental animals were performed under euthanasia, and all efforts were made to minimize suffering.

### Immunohistochemistry

Cultured BmN cells derived from silkworm ovaries and cultured in TC-100 medium (Gibco BRL, Rockville, MD, USA) at 27°C were collected and fixed with 4% paraformaldehyde for 15 min, then rinsed with 0.01 M PBST (0.05% of Tween-20 in PBS), and incubated with mouse anti-BmOVO-1 antiserum at 4°C overnight. As a negative control, BmN cells were treated with pre-immune antiserum. After rinsing with 0.01 M PBST three times, cells were incubated with FITC-conjugated goat anti-mouse IgG (Tiangen, Beijing, China) at 37°C for 1 h. Excess FITC-conjugated goat anti-mouse IgG was removed and cells were stained with DAPI and observed with a fluorescence microscope.

For histochemical observations, gonads were dissected from the larvae of the beginning and the end of the fifth instar larval stage, fixed in 4% paraformaldehyde and embedded in paraffin. Sections were processed with mouse anti-BmOVO-1 antiserum and TRITC-goat anti-mouse IgG (Tiangen, Beijing, China).

### Expression patterns of *Bmovo* genes

To analyze the *Bmovo* expression profile, cDNAs were synthesized by reverse transcription with 1 µg RNA extracted from tissues using PrimeScript Reverse Transcriptase. Primers for determining relative amounts of alternative spliced isoforms were designed based on cDNA sequences (Table S1). The *B*. *mori* housekeeping gene *actin A3* was used as an internal control and quantitatively amplified with primers semi-A3-F and semi-A3-R. Q-PCR used a real-time PCR System (Bio-Rad CFX96). Relative expression of *Bmovo* genes was estimated according to the 2^−ΔΔCt^ method [18].

### Reducing *Bmovo* in silkworms with RNAi

*Bmovo*-specific ovo-dsRNA-164 (sense: 5′-GCA CUG AUG GUG ACG UAU Utt-3′, antisense: 5′-AAU ACG UCA CCA UCA GUG Ctt-3′) was synthesized by Gima Corporation (Shanghai, China). A total of 40 silkworm larvae were injected with 2 µg ovo-dsRNA-164 per larva at the first day of the third instar stage and reared on fresh mulberry at 25°C. Expression of *Bmovo* in gonads of injected larvae at the first day of the fifth instar stage was estimated by Q-PCR. Negative control larvae were injected with random siRNA-NC (sense strand: 5′-UUC UCC GAA CGU GUC ACG Utt-3′; antisense strand: 5′-ACG UGA CAC GUU CGG AGA Att-3′). Blank controls were not injected and were raised normally.

### Producing and screening transgenic silkworms with upregulated *Bmovo*-1 (silkworm^+*Bmovo*-1^)

A 2.4 kb PCR product was amplified from plasmid pET28a(+)-Bmovo-1 using primers ovo-2/ovo-3 digested with *Xho*I and *Sal*I and ligated into plasmid piggyBacA3GFP to generate plasmid piggyBac-A3GFP-Bmovo-1. A promoter fragment from the *B. mori* vasa-like gene (*Bmvlg*) was amplified from plasmid pSK-vlg-DsRed-polyA [19] using primers Bmvlg-P1/Bmvlg-P3 and digested with *Sma*I and *Xho*I and cloned into piggyBacA3GFP-Bmovo-1 to generate the plasmid piggyBacA3GFP-VlgBmovo-1. A vlg-Bmovo-1 fragment from piggyBacA3GFP-VlgBmovo-1 digested with *Sma*I and *Xho*I was inserted into piggyBac-A3GFP-IeNeo [20] to obtain the plasmid piggyBac-A3GFP-IeNeo-VlgBmovo-1, in which a *Bmovo*-1 gene was driven by the Bmvlg promoter, and a neomycin gene (*neo*) was under control of the baculovirus immediately early gene (*ie*-1) promoter. The transgenic vector was confirmed with endonuclease digestion and sequencing.

Sperm-mediated gene transfer was performed as described by Zhou [21]. The transgenic vector piggyBac-A3GFP-IeNeo-VlgBmovo-1 at 1 µg/µL was mixed at a ratio of 1∶1 with a helper pigA3 vector containing the piggyBac transposase sequence under the control of the A3 promoter. The mixture was injected into the copulatory pouch of virgin moths (Strain Haoyue). Moths were free to copulate and oviposit. After spawning, embryos were incubated at 25°C at relative humidity 85–90% for 10 days until hatching. Larvae were reared on mulberry leaves at room temperature.

Newly hatched larvae were reared on mulberry leaves coated with 10 µg/mL G418 until approximately 10% remained. Surviving silkworms were observed under a stereomicroscope (Olympus SZX12, Tokyo, Japan) and fluorescent silkworms were fed normal mulberry leaves. Exogenous genes (*EGFP* and *NEO*) were identified with PCR from G2-generation transgenic silkworms using primers (DEGFP-1/DEGFP-2 and DENEO-1/DENEO-2). Genomic DNA extracted from fluorescent G2-generation moths was denatured by boiling and dotted onto nylon membranes. DNA hybridization was carried out with a DIG-labeled *gfp* probe. Membrane washing and signal detection used a DIG DNA labeling and detection kit (Roche, Mannheim, Germany), according to the manufacturer's instructions. Negative controls were genomic DNA from a normal silkworm; positive controls were plasmid pigA3GFP.

### Determination of trehalose content and trehalase activity assay

Trehalose contents of hemolymph and ovaries were estimated by the method of Ge *et al*. [22]. Trehalase activity in hemolymph was determined by the method of Yamashita *et al*. [23] and enzyme activities were expressed as µmol/ml hemolymph·h.

### Statistics

All data are presented as mean ± standard deviation (SD). The percentages of data were transformed into arcsine squareroot data for analysis. Statistical differences were evaluated using Student's *t*-test for unpaired samples.

## Results

### Cloning and characterization of the silkworm *Bmovo* gene

**Figure 1:**
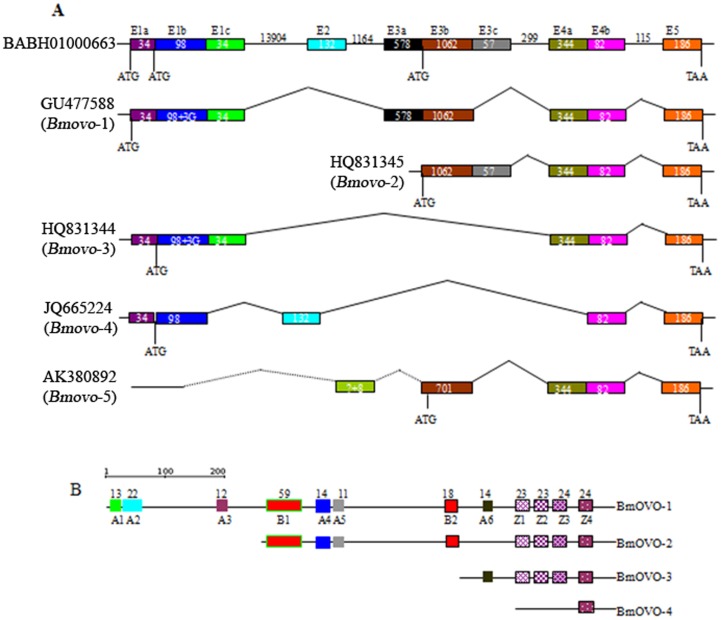
*Bmovo* gene structure and conserved domains in the deduced amino acid sequence. A: *Bmovo* gene structure and alternative splicing. Numbers indicate bp of exons and introns. Junctions of alternative spliced variants are marked. *Bmovo* is at contig BABH0100066 of the *B. mori* genome. E, exons, indicated with a box; lines, introns. B: Conserved domains in BmOVO. A, acidic region; B, basic region; Z, zinc-finger domain; numbers, number of amino acid residues in a domain.

To characterize the *Bmovo* gene, predicted gene sequences were obtained *in silico* based on DmOVO protein sequences. Three PCR products of 2.4, 0.8 and 0.5 kb were amplified from testes and ovary cDNA. Sequences from ovary and testes cDNA samples were same. The longest sequence was 2421 bp (GenBank accession No. GU477588) encoding a protein (BmOVO-1) with 806 amino acids. Two shorter sequences of 781 bp (GenBank accession No. HQ831344) and 498 bp (GenBank accession No. JQ665224) encoded BmOVO-3 with 248 (35–781 nt) and BmOVO-4 with 165 amino acids (Figure 1A). To detect alternatively spliced isoforms of *Bmovo*, primers Bmsovo-BH/Bmsovo-M2 and Bmsovo-M/Bmsovo-HD were used and specific 1.1 kb and 0.65 kb fragments were amplified from gonadal cDNA. Sequences of the products overlapped for an isoform of 1731 bp (GenBank accession No. HQ831345), encoding a protein (BmOVO-2) of 576 amino acids.

BlastN results showed that GenBank AK380892 had local identity with GU477588, HQ831344, HQ831345 and JQ665224 (Figure 1A). However, 8 nucleotides (taaatgaa) at the 5′-terminus of AK380892 were not found in GU477588, HQ831344, HQ831345 and JQ665224, suggesting that AK380892 was Bmovo-5, an alternatively spliced isoform of the Bmovo gene.

Comparison of the *Bmovo* gene cDNA sequence with the *B. mori* genome sequence using BlastN showed that *Bmovo* was in a genomic DNA fragment (BABH01000663) with 5 exons (Figure 1A). The longest isoform, *Bmovo*-1 (GU477588), had 4 exons, but exon 2 (E2) and E3c of exon 3 (E3) were not found. *Bmovo*-2 (HQ831345) was composed of partial codons from exons 3 (E3b and E3c), 4 (E4) and 5 (E5). *Bmovo*-3 (HQ831344) was composed of E1, exon 4 (E4) and E5, with the first ATG in E1b as the initiation codon. *Bmovo*-4 (JQ665224) was composed of E1a, E1b, E2, E4b and E5, with the first ATG in E1b as the initiation codon. AK380892, encoding 427 amino acids from a predicted start codon ATG at 38–40, had E4, E5 and partial codons of E3b, but the 5′ terminal sequence was unknown (Figure 1). The E1b sequence of GU477588/HQ831344 was identical to the corresponding region of genomic sequence BAAB01202273, but had three nucleotides that were different from genomic sequence BABH01000663 (Figure 1A). This result suggested that a genomic mutation resulted in E1b sequence differences between JQ665224 and GU477588/HQ831344.

Sequence comparisons showed that 61 amino acids were conserved at the carboxy-terminus among the five BmOVO isoforms and 203 amino acids were conserved at the carboxy-terminus among BmOVO-1, BmOVO-2, BmOVO-3 and BmOVO-4. Conserved domain search results showed that BmOVO-1, BmOVO-2 and BmOVO-3 had four common C2H2 zinc-finger domains (Z1–Z4), while BmOVO-4 had only one (Z4), indicating that BmOVO was possibly a transcription factor (Figure 1B).

BmOVOs differed at the N-termini, where effector domains were located. BmOVO-1 had six acidic regions (A1:13–25, pI 2.97; A2: 30–51, pI 4.08; A3: 180–191, pI 3.09; A4: 339–352, pI 3.20; A5: 362–372, pI 2.99; A6: 600–613, pI 3.09) and two basic regions (B1: 253–311, pI 12.20; B2: 540–557, pI 10.80) (Figure 1B). The four acidic regions A1, A2, A3 and A6 were not found in BmOVO-2. Four zinc-finger domains and the acidic region A6 were found in BmOVO-3. No known effectors were found in BmOVO-4. These results suggested that differences in conserved domains resulted in functional differences among the different BmOVO isoforms.

The deduced amino acid sequence of BmOVO-1 was aligned with the sequences of the other 5 isoforms. BmOVO-1 shared 52.2% similarity with OVO of *Apis mellifera* (XP_624482), 44.6% similarity with *Drosophila virilis* (XP_002057918), 47.9% similarity with *Pediculus humanus corporis* (XP_002423789), 50.8% similarity with *Solenopsis invicta* (EFZ10998), and 52.9% similarity with *Tribolium castaneum* (XP_974881). Although the sequence identities of OVO proteins from different species were not high, C2H2 zinc-finger domains were conserved (data not shown), suggesting that the DNA-binding sequences for OVO proteins were similar in different species.

Phylogenetic analysis by the neighbor-joining method suggested that *ovo* genes from different insect species clustered, while vertebrate genes formed another group. In insects, subgroups were the fruit fly *ovo* genes and *ovo* genes from silkworms and other insects. Although both fruit flies and mosquitoes belong to Diptera, their *ovo* genes were located on different branches (Figure S1).

### *Bmovo* expression patterns in silkworms

**Figure 2:**
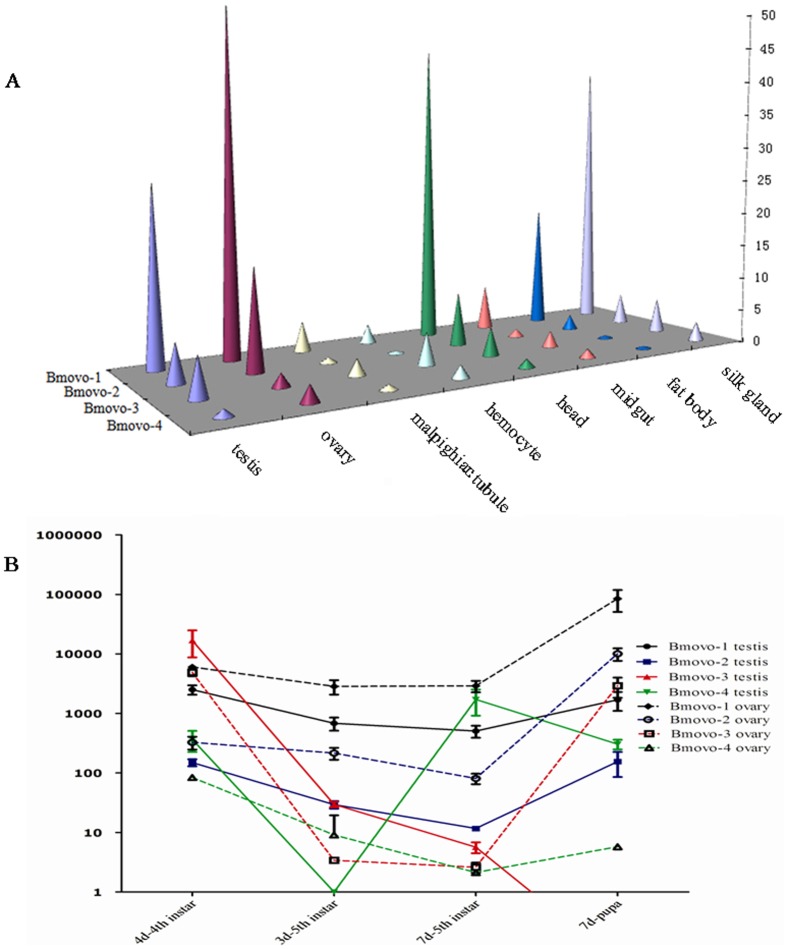
Expression profile of *Bmovo* genes in different tissues and various developmental stages by real-time PCR. A: Transcript levels of *Bmovo* relative to *Bmovo*-4 in tissues at day 3 of fifth instar stage. Square root transformation converted original data to data in the figure. B: Transcript levels of *Bmovo* relative to *Bmovo*-4 in the gonads at different stages: 4d-4th, day 4 of fourth instar; 3d-5th, day 3 of fifth instar; 7d-7th, day 7 of fifth instar; 7d-P, day 7 of pupal stage. All of the data were compared with the *Bmovo-4* gene from the day 3 of fifth instar. The *B*. *mori* housekeeping gene *actin A3* was used as an internal control. The values of the 2^−ΔΔCT^ were transformed into Log 10 data for analysis.

To investigate *Bmovo* gene expression patterns, 8 tissues were collected from fifth instar larvae on the third day. The highest expression of *Bmovo*-1 was in ovaries and was 3 times the expression in testes. *Bmovo*-1 was also highly expressed in the head and silk glands. The expression profile of *Bmovo*-2 was similar to the profile for *Bmovo*-1 in different tissues with the highest relative expression in ovaries. In contrast, expression of *Bmovo-*3 was 7.5 times higher than in the ovary. The highest expression of *Bmovo*-3 was in testes, then silk glands and hemocytes. *Bmovo*-4 was expressed at a lower level in different tissues in third-day of fifth instar silkworms; expression of *Bmovo*-4 was higher in silk glands than in ovaries (Figure 2A). Testes expression was highest for *Bmovo*-1 followed by *Bmovo*-2, *Bmovo*-3, and *Bmovo*-4. Expression of *Bmovo*-1 was 20 times higher in testes than *Bmovo*-2 or *Bmovo*-3. Ovarian expression was highest for *Bmovo*-1 followed by *Bmovo*-2, *Bmovo*-4, and *Bmovo*-3. Expression of *Bmovo*-1 was 11 times higher than expression of *Bmovo*-2, 450 times higher than *Bmovo*-4 and 708 times higher than *Bmovo*-3 (Figure 2B).

The four alternatively spliced isoforms of *Bmovo* were expressed differently at different developmental stages (Figure 2B). In testes, transcription of *Bmovo*-1 was highest at the fourth day of the fourth instar stage followed by the seventh day of the pupal stage. Transcriptional of *Bmovo*-1 at the fourth day of the fourth instar stage was 3 times higher than at the third and seventh days of the fifth instar stage. Compared to the seventh day of the fifth instar stage, transcription of *Bmovo*-2 was 13.96 times higher at the fourth day of the fourth instar stage and 11.38 times higher at the seventh day of the pupal stage. Compared to the seventh day of the fifth instar stage, transcription was 2 times higher at the third day of the fifth instar stage. Transcription of *Bmovo*-4 was the highest at the seventh day of the fifth instar stage and was 1715 times higher than that at the seventh day of the fifth instar stage, 3.5 times higher than the fourth day of the fourth instar stage and 4.4 times higher than the seventh day of the pupal stage. In the ovary, transcription of *Bmovo*-1 was the highest at the fourth day of the fourth instar stage, and was 2.2 times higher than at the third day of the fifth instar stage, 1.5 times higher than at the seventh day of the fifth instar stage, and 2.0 times higher than at the seventh day of the pupal stage. Transcription of *Bmovo*-2 was the highest at the seventh day of the pupal stage and was 19 times higher than at the fourth day of the fourth instar stage, 38 times higher than at the third day of the fifth instar stage and 96.8 times higher than at the seventh day of the fifth instar stage. Transcription of *Bmovo*-3 was the highest at the fourth day of the fourth instar stage and was 1432.2 times higher than at the third day of the fifth instar stage, 1883.5 times higher than at the seventh day of the fifth instar stage and 0.4 times higher than at the seventh day of the pupal stage. Transcription of *Bmovo*-4 was the highest at the fourth day of the fourth instar stage and was lower level at the third day of the fifth instar stage, the seventh day of the fifth instar stage and the seventh day of the pupal stage.

### BmOVO isoform analysis and immunohistochemistry

**Figure 3:**
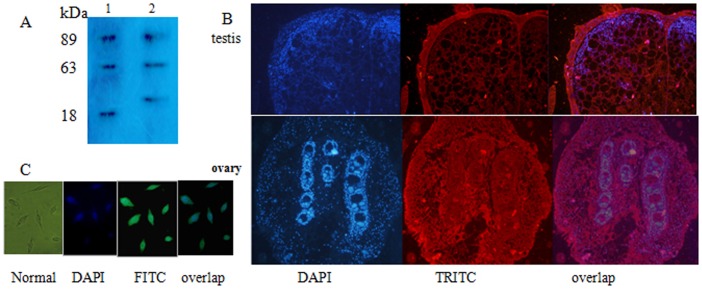
BmOVO in gonads and cultured BmN cells. A: SDS-PAGE and Western blotting of silkworm gonads at day 7 of 5th instar stage. lane 1, ovary; lane 2, testis. Primary antibody was mouse anti-BmOVO-1. Secondary antibody was HRP-conjugated goat anti-mouse IgG. Stacking gel 5% (v/v); separating gel, 10% (v/v). Proteins visualized chemoluminescence reagent. B: Immunofluorescence of gonads. Primary antibody was mouse anti-BmOVO-1 and secondary antibody was TRITC-conjugated goat anti-mouse IgG. C: Subcellullar location of BmOVO in cultured cells from ovaries. Primary antibody was mouse anti-BmOVO-1 and secondary antibody was FITC-conjugated goat anti-mouse IgG.

To examine the protein products from the *Bmovo* gene, total protein extracted from the testes and ovaries were subjected to SDS-PAGE and Western blotting. Specific protein bands of 89 kDa and 63 kDa representing BmOVO-1 and BmOVO-2, respectively, were detectable in both ovary and testis samples. A specific protein band of 18 kDa representing BmOVO-4 was detected only in the ovary samples and a specific protein band of 27 kDa representing BmOVO-3 was found only in testis samples (Figure 3A).

Immunofluorescence showed that BmOVO was expressed in follicular cells, nurse cells, primary oocytes and spermatids at different stages (Figure 3B). BmOVO in BmN cells was distributed in both the nucleus and cytoplasm, but mainly in nucleus (Figure 3C).

### Suppression of *Bmovo* expression in silkworms with *Bmovo*-siRNA

Expression of the *Bmovo* gene was reduced by injection of *Bmovo*-siRNA into silkworms at the third and fourth instar stage. In testes, *Bmovo*-1 transcription decreased 14% and *Bmovo*-2 transcription decreased 12% compared to the negative control. In ovaries of larvae in the first day of the fifth instar stage, *Bmovo*-1 transcription decreased 24% and *Bmovo*-2 transcription decreased 19% compared to the negative control. No difference was observed between the blank control and the negative control.

### Screening and identification of transgenic silkworms overexpressing *Bmovo*-1

**Figure 4:**
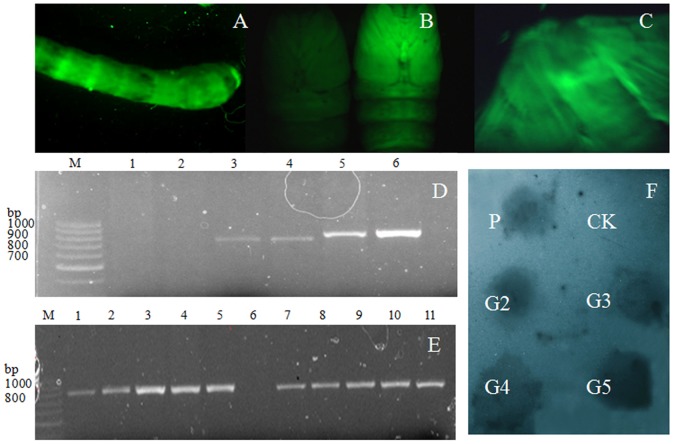
Screening and identification of transgenic silkworms. A: Fluorescent G2 larvae. B: Normal pupa (left), G2-generation transgenic pupa (right); C: fluorescent moth from G2-generation transgenic silkworms. D: PCR identification of G2-generation transgenic silkworms. M, DNA marker; lanes 1-2, PCR of normal silkworm genomic DNA with primers DEGFP-1/DEGFP-2 and primers DENeo-1/DENeo-2; lanes 3-4, PCR of GFP amplified from G2 fluorescent moths with primers pairs DEGFP-1/DEGFP-2; Lanes 5–6, PCR of neo amplified from fluorescent G2 moths generation with primers DENeo-1/DENeo-2. E: PCR identification of G3- and G4-generation silkworms using primers DENeo-1/DENeo-2. M, DNA marker; Lanes 1–4, PCR of genomic DNA of fluorescent silkworms (G3 generation); Lane 6, PCR of normal silkworm genomic DNA; Lane 7–11, PCR of genomic DNA from fluorescent silkworms (G4 generation); F: Identification of transgenic silkworm by dot hybridization with a DIG-labeled GFP probe. P, vector pigA3GFP; CK, genome extracted from normal silkworm; G2–G5, genome extracted from transgenic silkworms of G2–G5 generation.

A transgenic vector was mixed with helper plasmid and injected into copulatory pouches of six copulated female moths. Eggs were incubated at 25°C. Fluorescence was observed in some eggs, indicating that vector was introduced into the eggs. Transgenic silkworms exhibited neomycin resistance if the *neo* gene was successfully introduced into the genome and expressed. Larvae were reared on mulberry leaves coated with 10 mg/mL G418 from the second instar stage. Obvious developmental differences and deaths were observed among silkworms in response to G418. Fluorescence was observed in some surviving larvae (Figure 4A), pupae (Figure 4B) and moths (Figure 4C).

Genomic DNA of copulated moths was used amplify the *egfp* gene with primers DEGFP-1 and DEGFP-2. A 0.72 kb specific band with the *egfp* gene was amplified from fluorescent moths. G0 generation fluorescence moths were mated for a G1 generation. A G2 generation was generated by mutual crossing between fluorescent moths of generation G1. G3 and G4 generations were obtained in the same way. Fluorescent G2 silkworms were identified by PCR amplification using primer pairs DEGFP-1/DEGFP-2 and DENEO-1/DENEO-2. Specific bands for *egfp* (0.72 kb) and *neo* (0.8 kb) were detected in samples from fluorescent silkworms (Figure 4D). PCR products were purified and sequenced confirming *egfp* and *neo* genes in G2 moths and indicating that fluorescent G2 silkworms were transgenic. To verify that target genes were stably inherited in descendants of transgenic silkworms (silkworm^+*Bmovo*-1^), genomic DNA from G3 and G4 generation moths were amplified with primer pairs Neo-1/Neo-2 to detect products of *neo* gene (Figure 4E). These results indicated that heterogeneous genes were stably inherited in transgenic silkworms. Dot hybridization showed that a *egfp* probe hybridized with genomic DNA from fluorescent G2–G5 moths, indicating that the fluorescent silkworms were transgenic (Figure 4F).

To verify *Bmovo*-1 overexpression in silkworm^+*Bmovo*-1^, transcription of *Bmovo*-1 in the gonads of G5 generation silkworms at the third day of the fifth instar stage was estimated by Q-PCR. Transcription of *Bmovo*-1 in silkworm^+*Bmovo*-1^ was 2.265 higher in ovaries and 1.829 times higher in testes than in wild type silkworms.

### Impact of *Bmovo* on development of gonads, oviposition, cocoon yield and trehalose metabolism

**Figure 5:**
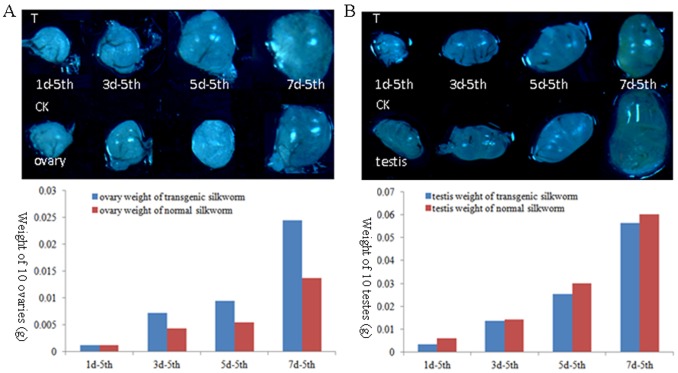
Effect of overexpressing *Bmovo*-1 gene on gonad weight. A: ovaries size display (up), ovaries weight statistics (down). B: teates size display (up), testes weight statistics (down). CK: ovaries collected from normal silkworm; T: ovaries collected from transgenic silkworm; 1d-5th, 3d-5th, 5d-5th and 7d-5th: day 1, 3, 5 and 7 of fifth instar stage.

Egg productivities per moth were 423 for the RNAi group, 505 for the negative control group and 506 for the normal group. Although egg productivity per moth in RNAi group was decreased by 16.40% compared with the normal group, disparity was not significant among the RNAi, negative control and normal groups for the rate of laid eggs. The rate of fertilized eggs was only slightly lower in the RNAi group than in the negative control and normal groups. However, the rates of hatched eggs from RNAi (♀) × RNAi (♂) and RNAi (♀) × normal (♂) were significantly lower than that from normal (♀) × RNAi (♂); no differences in hatched egg rates were observed from normal (♀) × RNAi (♂) (Table 1). Investigations of cocoon quantity from RNAi-injected silkworms showed increases of 10.47% for pupal weight and 11.16% for cocoon shell weight for females, and 0.96% and 10.79% for males compared to the blank control group (Table 2). In male silkworms in which the *Bmovo*-1 gene was upregulated in silkworm^+*Bmovo*-1^, pupal weight decreased by 11.28% and cocoon shell weight decreased by 22.04% in female silkworm^+*Bmovo*-1^ and decreased by 11.37% and 17.09% in G5 generation males (Table 3). In the G6 generation, pupal weight and cocoon shell weight were also decreased. The oviposition number of G6 generation silkworm^+*Bmovo*-1^ increased 13.37% compared to wild type silkworms (Table 4). Investigation of gonad development showed that ovary weight in silkworm^+*Bmovo*-1^ (G7 generation) worms was increased compared to wild type, but testes of silkworm^+*Bmovo*-1^ were less heavy than wild type (Figure 5A and 5B).

**Table 1:** Effect of silencing *Bmovo* on production traits.

Group		Female moth	Total egg productivity	Egg productivity per moth	Laid egg numbers per moth	Fertilized eggs per moth	Hatched eggs per moth	Rate of laid eggs (%)	Rate of fertilized eggs (%)	Rate of hatched eggs (%)
RNAi	RNAi(♀)×RNAi(♂)	7	3031	433a	430ab	416a	397Ab	99.31	96.74a	92.33AC
	RNAi(♀)×normal(♂)	5	2065	413ab	410ab	392a	352AB	99.27b	95.61A	89.80AB
	normal(♀)×RNAi(♂)	6	2916	486	486	467	458	100a	96.09A	98.07a
normal(♀) × normal(♂)		5	2545	509	508	505	498	99.80	99.41	98.61

Note: Characters (a, b, c) denote statistically significant differences compared with Group normal (♀) × normal(♂), normal (♀) ×RNAi (♂) and RNAi(♀) × normal(♂), respectively. Lowercase letters (a, b, c) represent *P*<0.05, capital letters (A, B, C) represent *P*<0.01.

**Table 2:** Effect of silencing *Bmovo* gene on pupa weight and cocoon shell weight.

Group	Sexuality	Pupa weight (g)	Cocoon shell weight (g)	Increase in pupa weight (%)	Increase in cocoon shell weight (%)
RNAi	male	0.5990	0.1000	0.96	10.79
	female	0.7518	0.0927	10.47	11.16
Negative control	male	0.5934	0.0904	0.02	0.12
	female	0.6834	0.0838	0.42	0.55
Blank control	male	0.5933	0.0903	-	-
	female	0.6806	0.0834	-	-

**Table 3:** Effect of overexpressing of *Bmovo*-1 on cocoon shell and pupa weight (*n*≥20).

Groups	Gender	Pupa weight (g)	Cocoon shell weight (g)	Descent range of pupa weight (%)	Descent range of cocoon shell weight (%)
G5 generation transformants	male	0.5263	0.0626	11.28	22.04
	female	0.6429	0.0621	11.37	17.09
Normal silkworms	male	0.5932	0.0803	-	-
	female	0.7254	0.0749	-	-
G6 generation transformants	male	0.6887	0.09838	13.2	21.2
	female	0.9116	0.1054	9.3	12.09
Normal silkworms	male	0.7937	0.1249	-	-
	female	1.0052	0.1199	-	-

**Table 4:** Effect of overexpressing *Bmovo*-1 on silkworm oviposition.

Groups	Number of female	Total produced egg numbers	Number of eggs laid per moth	Produced egg numbers per moth
Normal silkworms	6	1974	315	329
G6 generation transformants	8	2984	346	373

To understand nutrient resorption in different tissues, trehalose contents of hemolymph and ovaries at the fifth day of the fifth instar stage of silkworm^+*Bmovo*-1^ were estimated. Trehalose was 338.78 mg/100 ml in the hemolymph of wild type silkworms and 377.29 mg/100 ml in G7 generation silkworm^+*Bmovo*-1^. Trehalose was 3.06 mg/100 mg in ovaries of wild type silkworms and 3.39 mg/100 mg in G7 generation silkworm^+*Bmovo*-1^. Compared to wild type, trehalose was increased by 11.37% in the hemolymph and 10.56% in the ovaries of silkworm^+*Bmovo*-1^. Moreover, trehalase activity in hemolymph was also determined, but no significant difference was found between silkworm^+*Bmovo*-1^ and wild type (data not shown).

## Discussion

### Silkworm ovaries have different *Bmovo* transcripts and isoforms

In *B. mori*, the *ovo* gene region produces at least 4 transcripts and is different from the *D*. *melanogaster shavenbaby* (*svb*)-*ovo* gene region. The open reading frame (ORF) of the *Bmovo* gene consists of 5 exons. *Bmovo*-1 mRNA encodes only the BmOVO-1 isoform from the first AUG initiation codon in exon 1. BmOVO-3 and BmOVO-4 isoforms initiate from the second AUG initiation codon in exon 1 and the initiation codon for BmOVO-2 isoform is the first AUG in exon 3b. *D. melanogaster shavenbaby* (*svb*)-*ovo* has 5 annotated transcripts and 5 annotated polypeptides of 975, 1351, 1222, 1208 and 1351 amino acids. *Bmovo* has at least 4 transcripts and 4 predicted polypeptides of 806, 576, 248 and 165 amino acids. The molecular weight of BmOVO is lower than DmOVO.

*D*. *melanogaster* Svb/OVO isoforms contain four C2H2 zinc-finger motifs at the C-termini but have different N-termini that include potential effector domains. OVO-B and OVO-A are transcription factors with opposing regulatory activities [8]. The activation region contains a glycine-rich region (61% charged residues), two acidic regions (pI = 3.4, 3.7) and an extensive glutamine/histidine-rich region; the repression region contains a charged basic region (57% charged residues, pI = 11.9) and a serine-rich domain (46% charged residues) [4]. In *B*. *mori*, all BmOVOs have a common C2H2 zinc-finger domain, suggesting that BmOVO is a transcription factor that binds to promoters. BmOVO-1, BmOVO-2 and BmOVO-3 have four common C2H2 zinc-finger domains and BmOVO-4 has one. In *Drosophila*, OVO-B and OVO-A likely compete for similar binding sites. This might result in cross-regulation of target genes by the opposing activities of OVO-B and OVO-A [4]. The four BmOVO isoforms might also compete for binding sites and crossregulate expression of potential target genes.

BmOVOs differed at N-termini where the potential effector domains were located. BmOVO-1 had 6 acidic regions (A1–A6) and 2 basic regions (B1–B2); BmOVO-2 had 2 acidic regions (A4 and A5) and 2 basic regions (B1–B2) and BmOVO-3 had only an acidic region (A6). No effectors were found in BmOVO-4, suggesting that the difference in the conserved domain might result in a functional difference among the different BmOVO isoforms. The distribution and number of acidic and basic regions at the N-termini of BmOVO sequences differed from DmOVO, suggesting differences in function between BmOVO and DmOVO. In *Drosophila*, OVO-B and OVO-A are required for different developmental events; *Dmovo* is abundant in the ovary of adults and at the embryonic development stage of 00-02 hours [24]. Expression of GAL-tagged OVO proteins showed that overall OVO expression was strong during oogenesis, while OVO-A was expressed weakly and only in nearly mature follicles [2]. Consistent with this differential expression pattern, the germline-specific expression of *ovo* in females correlates with its function in oogenesis. This expression, however, is also seen in males that do not require *ovo* [2], [4].

### Expression pattern of *Bmovo* gene and its protein in silkworms

We found that in contrast to expression of *Dmovo*, *Bmovo* was expressed in several tissues of the fifth instar larvae of silkworms. The highest amounts of *Bmovo*-1 and *Bmovo*-2 mRNA were in ovaries. The highest amounts of *Bmovo*-3 were in testes. *Bmovo*-4 was mainly expressed in silk glands and ovaries, while *Dmovo*-B expression in larvae is restricted to gonads in both sexes [2]. These results suggested that BmOVO functions were not identical to DmOVO. Transcriptions of *Bmovo*-1, *Bmovo*-3 and *Bmovo*-4 were controlled by the same promoter but the transcripts had different AUG initiation codons, suggesting that differences in mRNA abundance of *Bmovo*-1, *Bmovo*-3 and *Bmovo*-4 might result from alternative splicing. In contrast to the expression pattern of *Bmovo*-1, *Bmovo*-3 and *Bmovo*-4, the expression pattern of *Bmovo*-2 might be determined by its own promoter. Western blotting showed that BmOVO-1, BmOVO-2 and BmOVO-4 were mainly expressed in ovaries, while BmOVO-3 was mainly expressed in testes. However, whether gonad development is influenced by the interaction of the 4 alternatively spliced isoforms is unclear.

The cysts of 64 primary spermatocysts emerge before the beginning of the third instar stage, then produce larger cysts of secondary spermatocysts from the fourth instar to the fifth instar stages, entering the second meiotic division that results in sperm bundles with mature sperm at the end of the fifth instar stage [25]. Q-PCR showed that transcription of the four *Bmovo* isoforms in testes changed with development. Levels of *Bmovo*-1, *Bmovo*-2 and *Bmovo*-3 at the fourth day of the fourth instar stage were higher than levels at the seventh day of the fifth instar stage. The highest transcription of *Bmovo*-4 was at the seventh day of the fifth instar stage, and the lowest transcription was at the third day of the fifth instar stage. *Ovo* expression is not required in *Drosophila* males [2], [4]. Therefore, the function of *Bmovo* in spermatogenesis of silkworms and *Drosophila* is worth further study.

### *Bmovo* impacting silkworm economic traits and production traits and trehalose metabolism

Tight regulation of antagonistic OVO-B and OVO-A isoforms is critical for germline formation and differentiation in *Drosophila* [4]. In *B*. *mori*, after *Bmovo*-1 and *Bmovo*-2 genes were silenced, the egg productivity per moth decreased by 16.40% compared with the control, suggesting that both *Bmovo*-1 and *Bmovo*-2 were required for oogenesis.

Although the rate of fertilized eggs was not different among the RNAi, negative control and normal groups, the rates of hatched eggs of both the RNAi (♀) × RNAi (♂) group and RNAi (♀) × normal (♂) group were significantly decreased compared with the control (Table 1), suggesting that repressing expression of *Bmovo*-1 and *Bmovo*-2 had little effect on spermatogenesis but influenced the development of embryogenesis and embryos. An investigation of cocoon quantity showed that pupal and cocoon shell weights in females increased and pupal weight in males did not change. However, cocoon shell weight increased in the RNAi group, indicating that silk protein synthesis was elevated by repressing *Bmovo*-1 and *Bmovo*-2.

**Figure 6:**
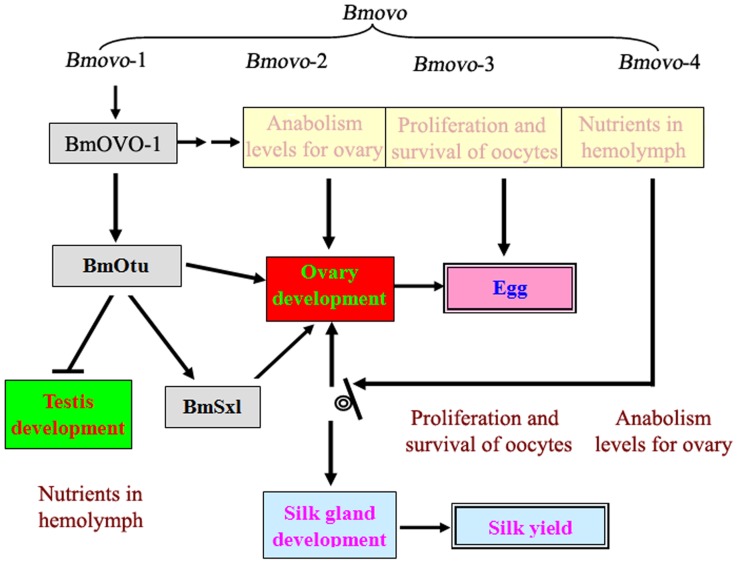
A model: *Bmovo*-1 gene regulation pathway.

The weight of both pupae and cocoon shells in silkworm^+*Bmovo*-1^ decreased compared with the control, while oviposition number increased by 13.37%. These data indicated that oogenesis and silk protein synthesis were influenced by regulating *Bmovo*-1 expression. Moreover, the gonadal weight was affected by overexpressing *Bmovo*-1 in silkworms. Ovary weight in silkworm^+*Bmovo*-1^ increased while testis weight decreased slightly, indicating that the function of BmOVO-1 was similar to the function of DmOVO-B. Genetic and molecular data indicated that *Dmovo* acted upstream of *otu^+^* and ultimately *Sxl^+^*. OVO-B positively regulated *otu* transcription in the female germline, suggesting that part of the function of OVO-B was upregulating Otu production. Maternal OVO-A was required for the progeny germline; OVO-A was extremely toxic when produced during early oogenesis. OVO-A repressed target genes expression is known to be part of the genetic hierarchy, including *ovo* [4]. BmOVO-1 acted similarly to OVO-B in *Drosophila* and might be a transcriptional activator that positively regulated *otu* and *sxl* transcription in the female silkworm germline. Overexpression of *Bmovo*-1 in silkworm gonads decreased *fib*-L transcription, reducing cocoon shell weight. Germline-specific expression of *ovo* in *Drosophila* females correlates with function in oogenesis. This expression, however, is also observed in males that do not require *ovo* [2], [4]. In silkworm^+*Bmovo*-1^, testis weight decreased slightly; nevertheless, the transcript levels from genes associated with spermatogenesis (*aly*, *achi*) were not significantly changed, suggesting that BmOVO-1 had no effect on spermatogenesis. Trehalose is a non-reducing disaccharide comprising two glucose molecules. It is the main haemolymph (blood) sugar with high concentrations presented in insects, which is hydrolysed by trehalase (E.C. 3.2.1.28) [26]. In silkworm^+*Bmovo*-1^, the trehalose content in the hemolymph and ovary were respectively elevated by 11.37% and 10.56%. In general, the ovary absorbs the nutrient from the hemolymph for egg development. *Bmovo*-1 overexpression in silkworm ovary may promote the transportation of lipid and/or sugar, which are conducive to ovary development and the excessive consumption of nutrient for ovary development altered nutrient partitioning and finally deters the silk protein synthesis (Figure 6).

## Supporting Information

Figure S1 **Phylogenetic tree based on OVO amino acid sequence.** Drm: *Drosophila melanogaster*; And: *Anopheles darlingi*; Ang: *Anopheles gambiae*; Apm: *Apis mellifera*; Bom: *Bombyx mori*; Peh: *Pediculus humanus corporis*; Soi: *Solenopsis invicta*; Trc: *Tribolium castaneum*; Dra: *Drosophila ananassae*; Dre: *Drosophila erecta*; Drs: *Drosophila sechellia Seychelles*; Drv: *Drosophila virilis*; Drw: *Drosophila willistoni*; Dry: *Drosophila yakuba*; Bao: *Bactrocera oleae*; Aim: *Ailuropoda melanoleuca*; Bot: *Bos taurus*; Caj: *Callithrix jacchus*; Caf: *Canis familiaris*; Gog: *Gorilla gorilla*; Hos: *Homo sapiens*; Mam: *Macaca mulatta*; Ora: *Ornithorhynchus anatinus*; Pat: *Pan troglodytes*; Ran: *Rattus norvegicus*; Mum: *Mus musculus*.
(TIF)

Table S1 **Primers used in this manuscript.**
(DOC)
