# Supplementary figures and images for: Bmovo-1 Regulates Ovary Size in the Silkworm, Bombyx mori
Source: PLoS One. 2014 Aug 13;9(8):e104928. doi: 10.1371/journal.pone.0104928 (PMC4132112; doi:10.1371/journal.pone.0104928)

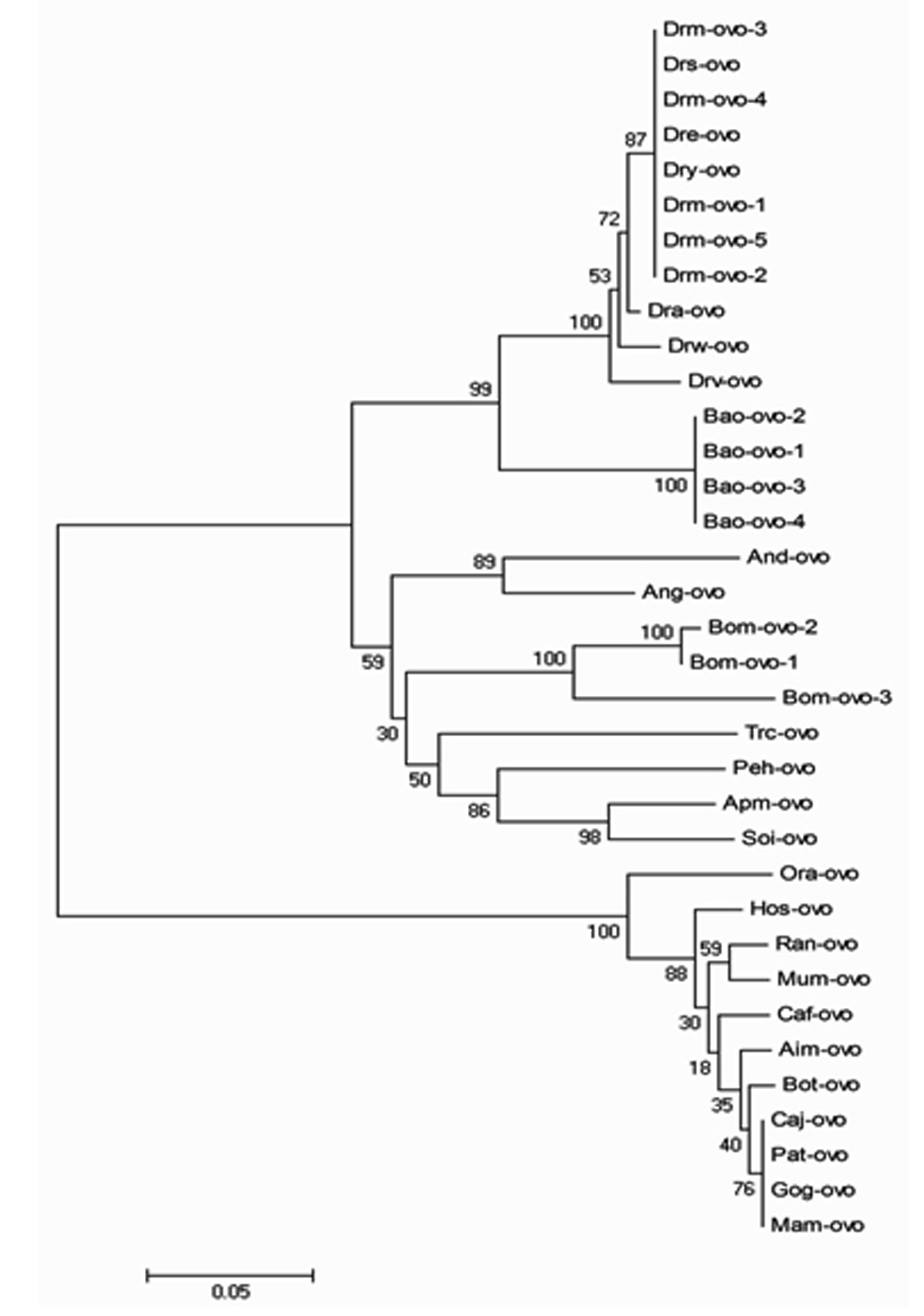

Supplement: Figure S1 — Phylogenetic tree based on OVO amino acid sequence. Drm: Drosophila melanogaster; And: Anopheles darlingi; Ang: Anopheles gambiae; Apm: Apis mellifera; Bom: Bombyx mori; Peh: Pediculus humanus corporis; Soi: Solenopsis invicta; Trc: Tribolium castaneum; Dra: Drosophila ananassae; Dre: Drosophila erecta; Drs: Drosophila sechellia Seychelles; Drv: Drosophila virilis; Drw: Drosophila willistoni; Dry: Drosophila yakuba; Bao: Bactrocera oleae; Aim: Ailuropoda melanoleuca; Bot: Bos taurus; Caj: Callithrix jacchus; Caf: Canis familiaris; Gog: Gorilla gorilla; Hos: Homo sapiens; Mam: Macaca mulatta; Ora: Ornithorhynchus anatinus; Pat: Pan troglodytes; Ran: Rattus norvegicus; Mum: Mus musculus. (TIF) [file pone.0104928.s001.tif]
