# Supplementary material for: Bmovo-1 Regulates Ovary Size in the Silkworm, Bombyx mori
Source: PLoS One. 2014 Aug 13;9(8):e104928. doi: 10.1371/journal.pone.0104928 (PMC4132112; doi:10.1371/journal.pone.0104928)
Supplement: Table S1 — Primers used in this manuscript. (DOC) [file pone.0104928.s002.doc]

Table S1 The primers used in this paper

| Primers | Sequences | recognition site | genes |
| --- | --- | --- | --- |
| ovo-1 | GGATCCATGCCGAAAATCTTCTGGATTAAG | *Bam*HI | Bmovo-1, 3 |
| ovo-2 | CTCGAGTTAATTGTGTACTGGCATGGGC | *Xho*I |
| BmSOVO-BH | TCCGGATCCATGTTAAATGCCGCTGCCG | *Bam*HI | Partial sequence of Bmovo-2 |
| BmSOVO-M2 | ATCTTTGTATCGGTGACAGAGC |  |
| BmSOVO-M | GCTCTGTCACCGATACAAAGAT |  | Partial sequence of Bmovo-2 |
| BmSOVO-HD | CGCAAGCTTAATTGTGTACTGGCATGGGC | *Hind*III |
| OVO1-HD | CGGAAGCTTATGCCGAAAATCTTCTGGATTAAG | *Hind*III | Bmovo-4 |
| OVO3-XH | CGGCTCGAGTTAATTGTGTACTGGCATGGGC | *Xho*I |
| OVO-3 | CTCGAGTTAATTGTGTACTGGCATGGGC | *Sal*I | Bmovo-1 |
| Bmvlg-P1 | CGCCCGGGGCCGCGCCGTAATCCTTCCACC | *Sma*I | Bmvlg promoter |
| Bmvlg-P3 | CGCTCGAGATTACCTGCAAAGTAATT | *Xho*I |
| semi-A3-F | CTGCGTCTGGACTTGGC |  | Actin A3 |
| semi-A3-R | CGAGGGAGCTGCTGGAT |  |
| semi-ovo-F | GCCCCTTACCGCTCCTTTCG |  | Bmovo-1 |
| semi-ovo-R | ATCGCCTCCAAGAATCGATG |  |
| DEGFP1 | 5’-TGGAATTCATGGTGAGCAAGGGCGAGG-3’ |  | gfp |
| DEGFP2 | 5’-TTGGATCCTTACTTGTACAGCTCGTCCATG-3’ |  |
| DENEO- 1 | GAATTCATGATTGAACAAGATGGATTGCACG |  | neo |
| DENEO- 2 | GGATCCTCAGAAGAACTCGTCAAGAAGGCG |  |
